# Causal relationship between sex hormones and ischemic stroke risk: A Mendelian randomization study

**DOI:** 10.1097/MD.0000000000050336

**Published:** 2026-09-04

**Authors:** Qiang Huang, Qiong Li, Jun-Hong Guo

**Affiliations:** a Department of Neurology, The First People’s Hospital of Jinzhong, Jinzhong, Shanxi, China; b Department of Endocrinology, The First People’s Hospital of Jinzhong, Jinzhong, Shanxi, China; c Department of Neurology, First Hospital of Shanxi Medical University, Taiyuan, Shanxi, China.

**Keywords:** causal inference, ischemic stroke, Mendelian randomization, sex hormones, SHBG

## Abstract

Ischemic stroke (IS) exhibits notable sex-related differences, indicating a potentially important role for sex hormones such as sex hormone–binding globulin (SHBG), estradiol, and testosterone. This study employed 2-sample Mendelian randomization (MR) to investigate the causal relationships between these sex hormones and the risk of IS and its subtypes. Genetic instrumental variables for SHBG, estradiol, and testosterone were obtained from the IEU OpenGWAS database. These instruments were analyzed to assess their causal effects on IS and its subtypes using MR methods. Sensitivity analyses were performed using several complementary MR approaches. SHBG was inversely associated with the risk of IS (OR = 0.92; 95% CI = 0.87–0.97; *P* = .007) and with the small-artery occlusion subtype (OR = 0.84; 95% CI = 0.75–0.94; *P* = .002), whereas no associations were observed for the large-artery atherosclerosis or cardioembolism subtypes. No causal effects of estradiol or testosterone on IS were identified. Our findings suggest that higher SHBG levels may reduce the risk of IS and could represent a potential therapeutic target. Further studies are needed to elucidate the mechanisms by which SHBG may confer protection against IS.

## 1. Introduction

Stroke is the second leading cause of death worldwide, following cancer, with ischemic stroke (IS) being the most prevalent subtype and posing substantial socioeconomic and healthcare burdens. Marked sex differences have been observed in both the prevalence and severity of stroke.^[1]^ Epidemiological evidence indicates that with advancing age, the incidence of stroke in women surpasses that in men, and their recovery outcomes tend to be poorer. Furthermore, premenopausal women exhibit a lower incidence of stroke than men; however, after menopause, the incidence in women increases to approximately twice that observed in men.^[2]^ These sex differences suggest a potential relationship between stroke risk and sex hormone levels. Testosterone and estradiol, as key sex hormones, have been associated with stroke risk in several observational studies.^[3-8]^ Sex hormone–binding globulin (SHBG), a liver-derived protein, regulates the bioavailability of sex hormones by binding to them and thus plays an essential role in hormone-related physiological processes.^[9]^

Although numerous studies have reported associations between sex hormones and stroke,^[1,2]^ many lack high-quality evidence, making their conclusions difficult to interpret or standardize. Moreover, conducting randomized controlled trials (RCTs) in this field is often impractical due to the substantial human, material, and financial resources required. Consequently, these study designs are unable to fully eliminate reverse causality and confounding.

Mendelian randomization (MR) has emerged as a powerful approach to inferring causal relationships between risk factors and diseases. MR leverages the random assortment of genetic variants during meiosis, using single-nucleotide polymorphisms (SNPs) as instrumental variables to estimate causal effects between exposures and outcomes. Because genetic variants are randomly allocated at conception and precede disease onset, MR analyses can substantially reduce confounding and reverse causation, thereby strengthening causal inference.

In this study, we systematically evaluated the causal relationships between sex hormones (SHBG, estradiol, and testosterone) and IS and its subtypes using MR methods.

## 2. Materials and methods

### 2.1. Study design and data sources

**Figure 1.**
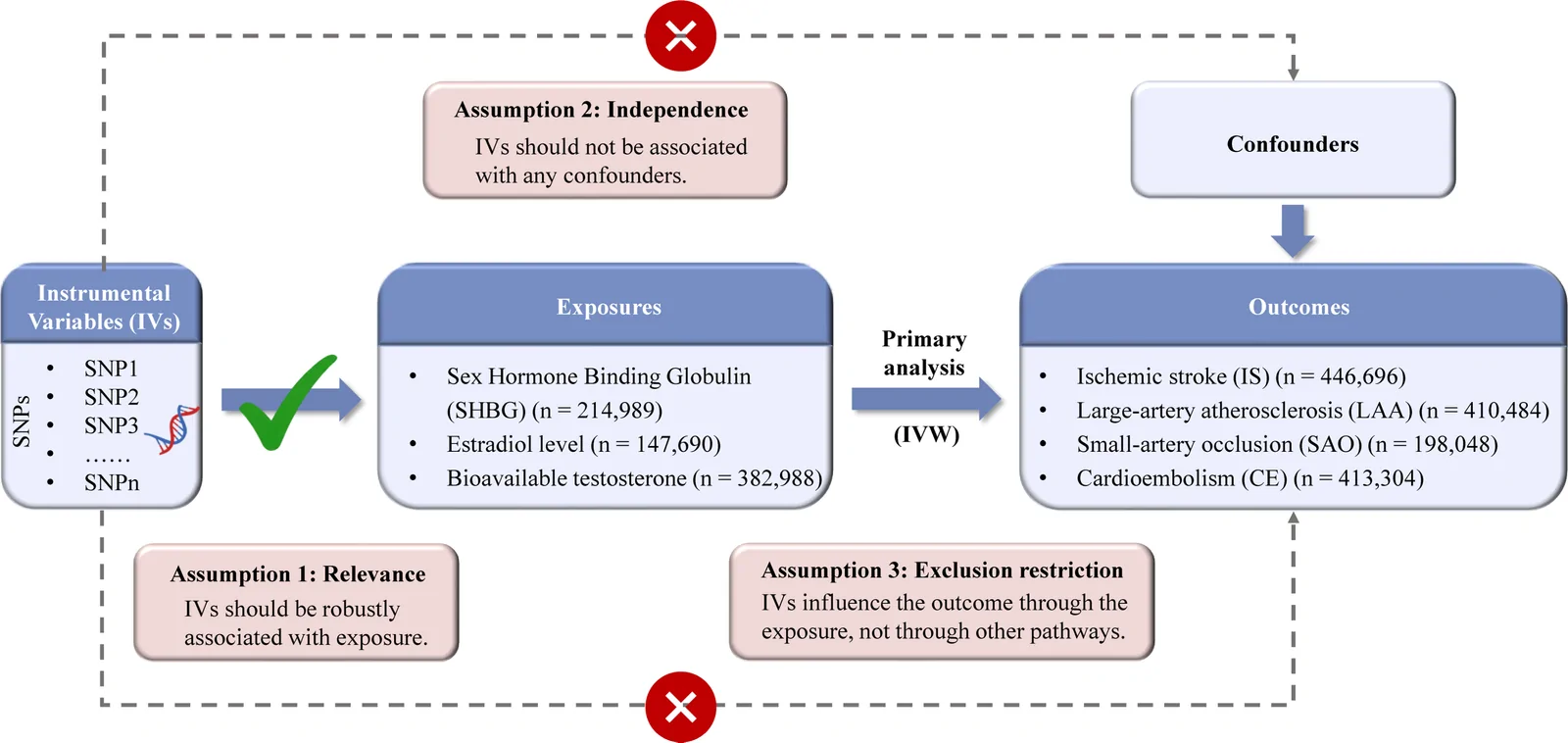
Study design for this Mendelian randomization analysis. CE = cardioembolism, IS = ischemic stroke, LAA = large artery atherosclerosis, SAO = small-artery occlusion, SHBG = sex hormone–binding globulin, SNP = single-nucleotide polymorphisms.

This study employed a 2-sample Mendelian randomization (MR) approach to evaluate the causal relationships between genetically predicted sex hormone levels and ischemic stroke (IS), including its subtypes – large artery atherosclerosis (LAA), small-artery occlusion (SAO), and cardioembolism (CE). Exposure and outcome data were obtained from nonoverlapping datasets (Table S1, Supplemental Digital Content 1), both derived from European populations. The MR design adhered to 3 core assumptions: the genetic instruments were strongly associated with the exposures (*P* < 5 × 10^−8^); the instruments were not associated with confounders; and the instruments influenced the outcomes exclusively through the exposures (Fig. 1). The included exposure-SNPs were presented in Table S2–S4, Supplemental Digital Content 2. The study was conducted according to the STROBE-MR-checklist as provided in Supplementary materials.

Data for sex hormones, including SHBG, estradiol, and testosterone, were obtained from the IEU OpenGWAS database (https://gwas.mrcieu.ac.uk/). SHBG data were derived from a European cohort of 2,14,989 individuals, estradiol data from 1,47,690 individuals, and testosterone data from 3,82,988 individuals.

Data for IS and its subtypes were also obtained from the IEU OpenGWAS database.^[9]^ Specifically, IS data comprised 40,585 cases and 4,06,111 controls from a European population; LAA data comprised 4373 cases and 4,06,111 controls; SAO data comprised 5386 cases and 1,92,662 controls; and CE data comprised 7193 cases and 4,06,111 controls.

All data used in this study were obtained from publicly accessible databases, and ethical approval was secured in the original studies.

### 2.2. Selection of genetic instrumental variables

Genetic instrumental variables (IVs) for SHBG, estradiol, and testosterone were obtained from the IEU OpenGWAS database. A total of 197 independent single-nucleotide polymorphisms (SNPs) associated with SHBG were extracted (*P* < 5 × 10^−8^) using linkage disequilibrium (LD) clumping criteria (*r*^2^ < 0.001).^[10]^ For IS and SAO, 20 and 13 SNPs, respectively, were excluded due to evidence of genetic heterogeneity. Exposure-related IVs were selected based on the following criteria: genome-wide significance (*P* < 5 × 10^−8^); LD clumping using the PLINK algorithm (*r*^2^ < 0.001); and exclusion of SNPs with evidence of potential pleiotropy. Additionally, 13 independent SNPs associated with estradiol and 124 SNPs associated with bioavailable testosterone were identified using the same criteria.

### 2.3. Statistical analysis

This study employed 5 methods for Mendelian randomization (MR) analysis: inverse variance weighted (IVW), MR-Egger, weighted median, simple mode, and weighted mode. IVW was used as the primary analytical approach to assess the causal relationships between sex hormones and IS and its subtypes. The MR-Egger method incorporates an intercept term to account for the average directional pleiotropic effects of all genetic variants.^[11]^ To further evaluate and mitigate the impact of horizontal pleiotropy on causal estimates, we applied the MR-PRESSO framework, which detects and corrects for outlier instrumental variables.^[12]^

Cochran *Q*-test was used to assess heterogeneity, with statistical significance defined as *P* < .05. Leave-one-out analyses were performed to examine whether the causal estimates were driven by any single SNP by sequentially excluding each SNP and rerunning the IVW analysis. To correct for multiple testing, Bonferroni adjustments were applied, with statistical significance set at *P* < .017 (0.050/3). *P*-values between .017 and .050 were considered suggestive of potential statistical significance.^[13]^

To detect the direct impact of sex hormones on the risk of stroke, we also conducted a multivariable MR (MVMR) analysis taking low-density lipid (LDL) into account. Specifically, MVMR is the extension of traditional 2-sample MR analysis. Although a series of sensitivity analyses were used to detect the validity of the MR results, residual pleiotropy or confounding could not be ruled out. Thereby, MVMR was used to adjust the LDL level when evaluating the causal effect of sex hormones on the risk of stroke. The SNPs used for LDL came from Richardson et al’s GWAS study.^[14]^ The parameters for proceeding SNP filtering were as mentioned above.

All statistical analyses were conducted using R software (version 4.3.1) and relevant R packages, including “MendelianRandomization”^[15]^ and “MR-PRESSO”^[12]^ packages.

## 3. Results

### 3.1. Instrumental variables in this study

After data harmonization, 167 SNPs for IS, 169 SNPs for LAA, 148 SNPs for SAO, and 172 SNPs for CE were selected as genetic instruments for the MR analyses of SHBG in relation to IS and its subtypes. For the MR analyses of estradiol in relation to IS and its subtypes, 8 SNPs for IS, 9 SNPs for LAA, 7 SNPs for SAO, and 10 SNPs for CE were selected as genetic instruments. Similarly, 94 SNPs for IS, 96 SNPs for LAA, 83 SNPs for SAO, and 96 SNPs for CE were selected for the MR analyses of bioavailable testosterone in relation to IS and its subtypes.

### 3.2. SHBG and ischemic stroke and its subtypes

**Figure 2.**
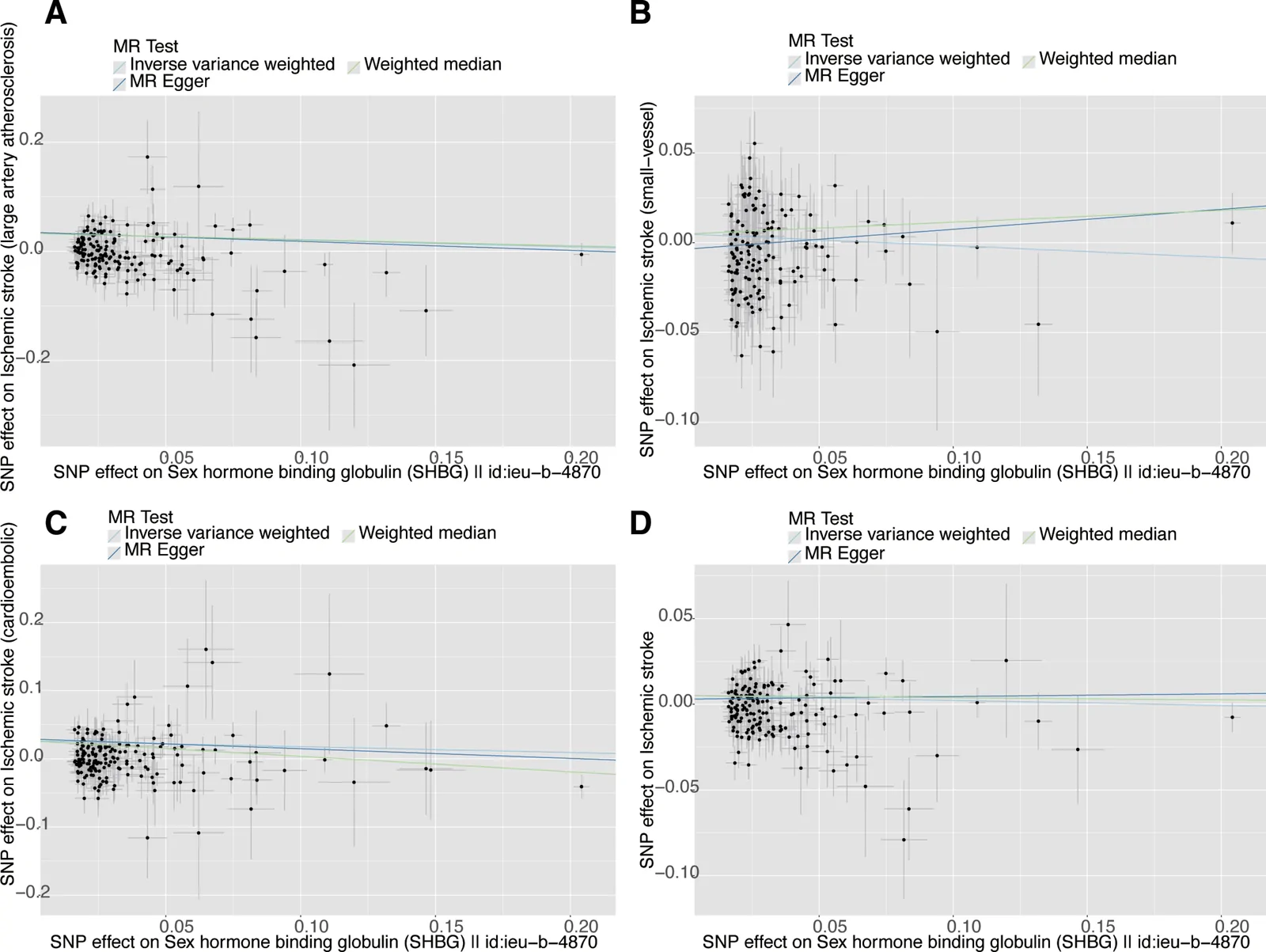
Scatter plot of the causal effects of SHBG on the risk of LAA (A), SAO (B), CE (C), and IS (D). CE = cardioembolism, IS = ischemic stroke, LAA = large artery atherosclerosis, MR = Mendelian randomization, SAO = small-artery occlusion, SHBG = sex hormone–binding globulin, SNP = single-nucleotide polymorphisms.

Table 1 and Figure 2D show a negative causal effect of SHBG on IS based on the IVW method (OR = 0.925; 95% CI: 0.874–0.979; *P* = .007). However, the weighted median, simple mode, and weighted mode analyses did not support a causal relationship between SHBG and IS risk (Table 1). The MR-Egger method provided no evidence of directional pleiotropy among the instrumental variables. Owing to heterogeneity, the RadialMR method identified 20 SNP outliers. As shown in Table 1 and Figure 2B, a negative causal effect of SHBG on SAO was detected (OR = 0.844; 95% CI: 0.756–0.942; *P* = .002). However, similar to the findings for IS, the weighted median, simple mode, and weighted mode analyses did not support this causal relationship (Table 1). The MR-Egger method again showed no evidence of directional pleiotropy. No causal relationships were observed between SHBG and the risks of LAA or CE (Table 1and Fig 2A, C). However, when adjusted for LDL levels, the impact of SHBG on the risk of stroke vanished (OR = 0.98, 95% CI: 0.93–1.03, *P* = .34).

**Table 1:** Mendelian randomization analysis results for the causal associations of sex hormones (SHBG, estradiol) with IS, LAA, SAO, and CE.

Method			SHBG				Estradiol	
	SNPS (N)	OR	95% CI	*P*-value	SNPS (N)	OR	95% CI	*P*-value
IS								
IVW	167	0.925	0.874–0.979	.007	8	1.038	0.981–1.097	.186
Weighted median	167	0.933	0.855–1.019	.096	8	1.036	0.972–1.105	.273
MR Egger	167	0.952	0.854–1.062	.385	8	1.097	0.936–1.285	.292
Simple mode	167	0.894	0.747–1.071	.228	8	1.030	0.926–1.145	.595
Weighted mode	167	0.930	0.837–1.034	.183	8	1.031	0.955–1.113	.455
LAA								
IVW	169	0.948	0.840–1.071	.399	9	1.043	−0.091 to 0.175	.535
Weighted median	169	0.958	0.778–1.181	.692	9	1.063	−0.097 to 0.219	.448
MR Egger	169	0.868	0.686–1.099	.243	9	1.049	−0.328 to 0.424	.809
Simple mode	169	0.728	0.463–1.144	.170	9	1.044	0.801–1.359	.756
Weighted mode	169	0.854	0.651–1.120	.256	9	1.062	0.898–1.256	.496
SAO								
IVW	148	0.844	0.756–0.942	.002	7	0.987	0.880–1.108	.835
Weighted median	148	0.882	0.740–1.051	.162	7	0.950	0.831–1.087	.459
MR Egger	148	0.989	0.788–1.241	.924	7	0.852	0.615–1.180	.381
Simple mode	148	0.792	0.540–1.161	.234	7	0.969	0.775–1.213	.796
Weighted mode	148	0.880	0.710–1.089	.243	7	0.944	0.816–1.093	.477
CE								
IVW	172	0.971	0.874–1.079	.592	10	0.978	0.807–1.185	.822
Weighted median	172	0.894	0.765–1.046	.164	10	0.913	0.792–1.052	.211
MR Egger	172	0.884	0.725–1.078	.255	10	0.995	0.570–1.738	.988
Simple mode	172	0.823	0.539–1.256	.368	10	0.943	0.651–1.367	.765
Weighted mode	172	0.778	0.591–1.025	.076	10	0.907	0.780–1.054	.236

CE = cardioembolism, CI = confidence interval, IS = ischemic stroke, IVW = inverse variance weighted, LAA = large artery atherosclerosis, MR = Mendelian randomization, OR = odds ratio, SAO = small-artery occlusion, SNP = single-nucleotide polymorphisms, SHBG = sex hormone–binding globulin.

### 3.3. Estradiol and ischemic stroke and its subtypes

**Figure 3.**
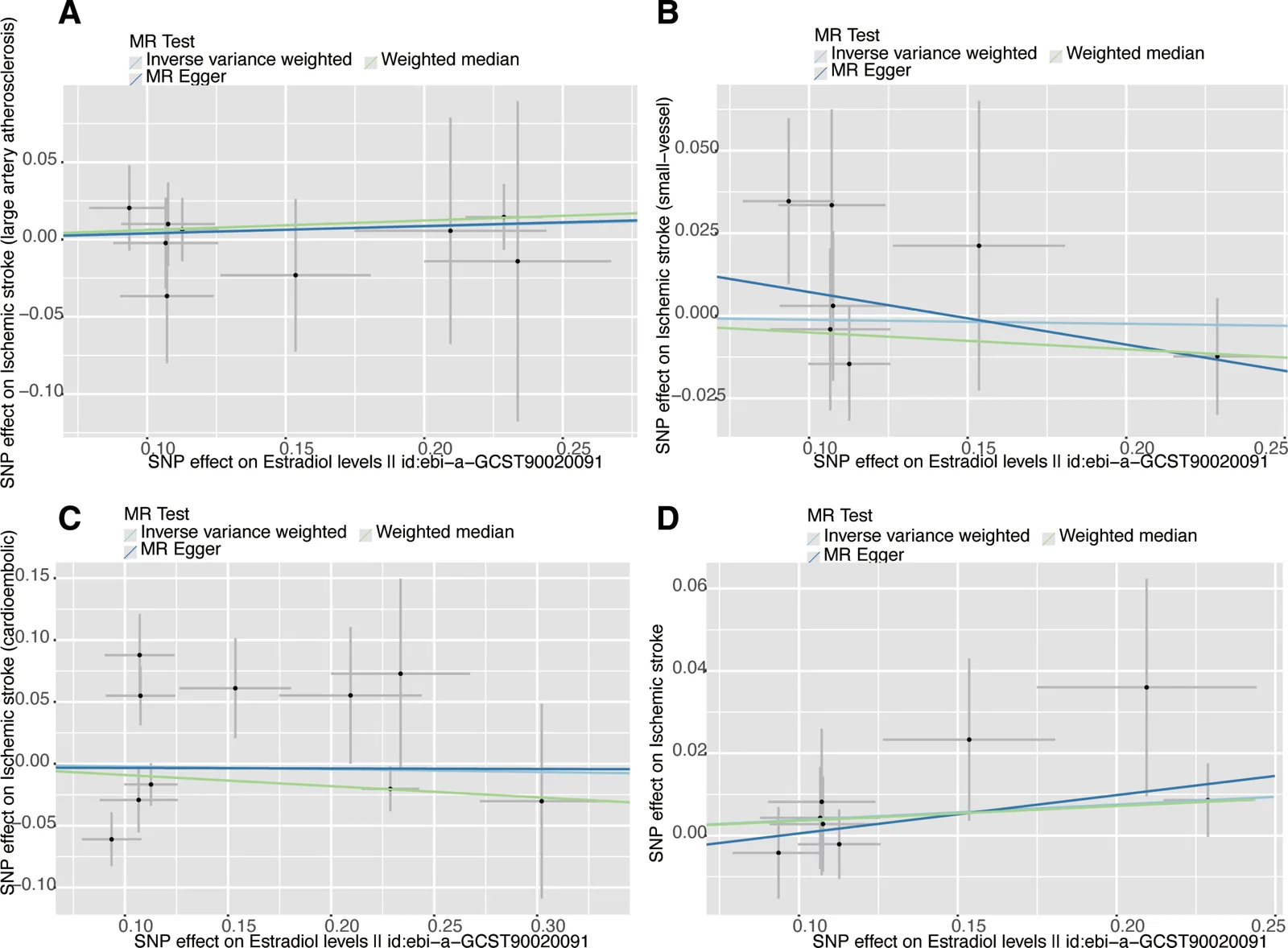
Scatter plot of the causal effects of estradiol levels on the risk of LAA (A), SAO (B), CE (C), and IS (D). CE = cardioembolism, IS = ischemic stroke, LAA = large artery atherosclerosis, MR = Mendelian randomization, SAO = small-artery occlusion, SNP = single-nucleotide polymorphisms.

As shown in Table 1 and Figure 3A–D, the IVW analysis did not detect a genetically causal relationship between estradiol and IS (*P* = .186) or its subtypes, including LAA (*P* = .535), SAO (*P* = .835), and CE (*P* = .822). Similarly, other MR analytical methods also did not reveal any causal associations between estradiol and IS or its subtypes (Table 1).

### 3.4. Bioavailable testosterone and ischemic stroke and its subtypes

**Figure 4.**
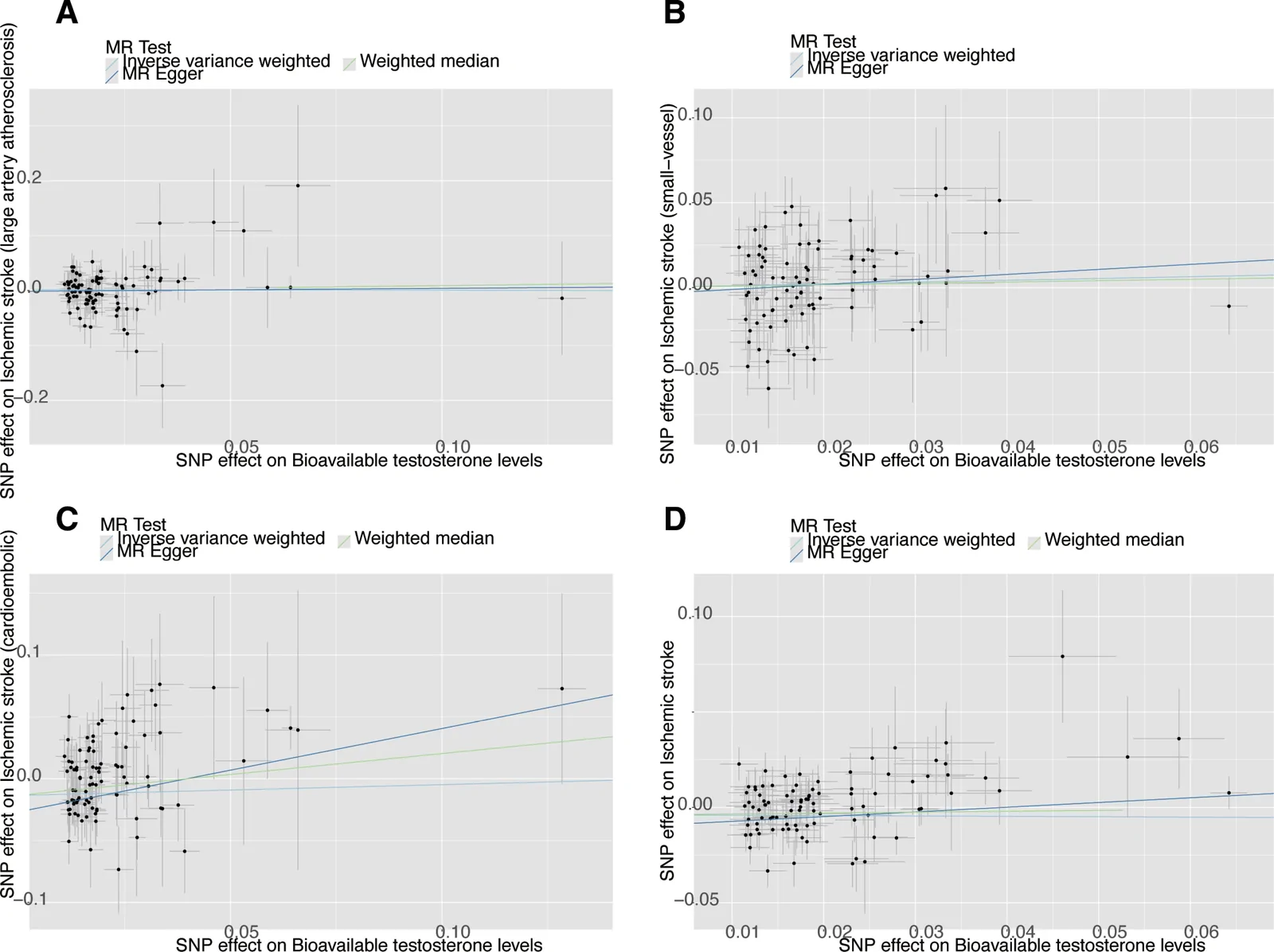
Scatter plot of the causal effects of bioavailable testosterone levels on the risk of LAA (A), SAO (B), CE (C), and IS (D). CE = cardioembolism, IS = ischemic stroke, LAA = large artery atherosclerosis, MR = Mendelian randomization, SAO = small-artery occlusion, SNP = single-nucleotide polymorphisms.

Table 2 and Figure 4A–D show that the IVW analysis did not detect any genetically causal relationships between bioavailable testosterone and IS (*P* = .475) or its subtypes, including LAA (*P* = .970), SAO (*P* = .429), and CE (*P* = .779). Likewise, other MR analytical methods also did not identify any causal associations between bioavailable testosterone and IS or its subtypes (Table 2).

**Table 2:** Mendelian randomization analysis results for the causal associations of sex hormones (SHBG, bioavailable testosterone) with IS, LAA, SAO, and CE.

Method			SHBG				Bioavailable testosterone	
	SNPS (N)	OR	95% CI	*P*-value	SNPS (N)	OR	95% CI	*P*-value
IS								
IVW	167	0.925	0.874–0.979	.007	94	1.045	0.925–1.180	.475
Weighted median	167	0.933	0.855–1.019	.096	94	1.122	0.922–1.366	.249
MR Egger	167	0.952	0.854–1.062	.385	94	1.359	1.020–1.811	.038
Simple mode	167	0.894	0.747–1.071	.228	94	1.317	0.885–1.959	.177
Weighted mode	167	0.930	0.837–1.034	.183	94	1.156	0.937–1.426	.178
LAA								
IVW	169	0.948	0.840–1.071	.399	96	1.004	0.776–1.301	.970
Weighted median	169	0.958	0.778–1.181	.692	96	1.093	0.683–1.749	.710
MR Egger	169	0.868	0.686–1.099	.243	96	1.032	0.567–1.880	.916
Simple mode	169	0.728	0.463–1.144	.170	96	1.352	0.554–3.298	.508
Weighted mode	169	0.854	0.651–1.120	.256	96	1.199	0.732–1.965	.471
SAO								
IVW	148	0.844	0.756–0.942	.002	83	1.101	0.866–1.399	.429
Weighted median	148	0.882	0.740–1.051	.162	83	1.079	0.756–1.540	.672
MR Egger	148	0.989	0.788–1.241	.924	83	1.326	0.741–2.371	.344
Simple mode	148	0.792	0.540–1.161	.234	83	1.483	0.688–3.194	.316
Weighted mode	148	0.880	0.710–1.089	.243	83	0.942	0.620–1.431	.780
CE								
IVW	172	0.971	0.874–1.079	.592	96	1.036	0.807–1.330	.779
Weighted median	172	0.894	0.765–1.046	.164	96	1.350	0.962–1.894	.082
MR Egger	172	0.884	0.725–1.078	.255	96	1.830	1.049–3.191	.035
Simple mode	172	0.823	0.539–1.256	.368	96	1.540	0.635–3.732	.341
Weighted mode	172	0.778	0.591–1.025	.076	96	1.540	0.986–2.404	.060

CE = cardioembolism, CI = confidence interval, IS = ischemic stroke, IVW = inverse variance weighted, LAA = large artery atherosclerosis, MR = Mendelian randomization, OR = odds ratio, SAO = small-artery occlusion, SHBG = sex hormone–binding globulin, SNP = single-nucleotide polymorphisms.

## 4. Discussion

This study systematically investigates the causal relationships between sex hormones and IS and its subtypes, thereby enriching the existing body of research. Our findings suggest a potential causal relationship between SHBG and both IS and SAO, whereas no causal associations were observed between estradiol, testosterone, and IS or its subtypes. IS is a common neurological condition among the elderly, and numerous studies have suggested associations between sex hormones and IS.^[16]^However, these studies lacked systematic approaches, seldom explored the associations between IS subtypes and sex hormones, and were predominantly based on observational cohorts with limited causal evidence. In this study, we demonstrated robust causal relationships between SHBG and both IS and SAO. Elevated SHBG levels are associated with reduced concentrations of biologically active testosterone. Testosterone deficiency may accelerate vascular aging through mechanisms involving oxidative stress and inflammation, potentially contributing to cerebrovascular disease.^[7,17]^ However, the role of androgens in the cerebrovascular system remains unclear and may vary depending on factors such as sex, age, dosage, and other biological variables.^[18]^ For instance, although androgens are pro-inflammatory under physiological conditions, they exhibit anti-inflammatory effects in pathological states.^[18]^ Therefore, the precise mechanisms through which SHBG reduces the risk of IS and its subtypes warrant further investigation.

Additionally, our findings did not reveal any clear causal relationships between estradiol, testosterone, and IS or its subtypes. Previous studies have highlighted the dynamic nature of the associations between these hormones and IS, emphasizing the influence of factors such as age and sex.^[18]^ Randomized controlled trials (RCTs) examining estrogen-related factors – such as age at menopause, hormone replacement therapy, oral contraceptive use, pregnancy, and hysterectomy – have often produced contradictory results due to small sample sizes and confounding variables.^[19,20]^ Further research is needed to enhance our understanding of these complex interactions.

To the best of our knowledge, this is the first MR study to investigate the relationships between sex hormones and the risk of IS. MR studies offer significant advantages over traditional observational designs by reducing confounding and minimizing the risk of reverse causality.^[21]^ The application of multiple MR methods, together with recently published GWAS data and extensive sensitivity analyses, strengthened the robustness of our findings. Notably, 2 recent studies using MR techniques explored the association of serum SHBG levels with the risk of ischemic stroke.^[22,23]^ Both of them focused only on SHBG. Our study systematically analyzed distinct sex hormones on the risk of ischemic stroke and its subtypes. In addition, we also conducted MVMR to rule out the potential influence of LDL, making the causal inference more convincing.

However, this study has several limitations. First, because the study was conducted among participants of European ancestry, the findings may not be generalizable to other populations with different lifestyles and cultural backgrounds. Although our 2-sample MR analysis used data from different sources, which may introduce heterogeneity, we addressed this issue by excluding outliers exhibiting substantial heterogeneity. Sensitivity analyses revealed no substantial pleiotropy in our results. Second, the potential mechanisms from SHBG to decreased risk of stroke warrant future analysis, including investigating the interaction among SHBG and its downstream molecules using molecular docking, molecular dynamics simulations, and so on. This exploratory study aims to provide evidence for the potential impact of SHBG on the risk of stroke, which would help guide future experimental study on the mechanisms of the development of stroke. Third, given that gender- and age-stratified GWAS was not available, stratification analysis in this MR work was not allowed. Future investigation into the impact of SHBG on the risk of stroke in specific populations is warranted when stratification GWAS becomes publicly available.

## 5. Conclusion

This study is the first to demonstrate a causal relationship between SHBG levels and the risk of ischemic stroke (IS), particularly the small artery occlusion (SAO) subtype. However, no causal relationship was identified between testosterone, estradiol, and IS or any of its subtypes. These findings underscore the potential importance of monitoring SHBG levels in the general population as a strategy for early IS prevention. Further research is warranted to elucidate the biological mechanisms underlying this causal relationship, which should be investigated in follow-up experimental studies.

## Acknowledgments

We are grateful for the IEU database for making GWAS data publicly available.

## Author contributions

**Data curation:** Qiong Li.

**Methodology:** Jun-Hong Guo.

**Writing – original draft:** Qiang Huang.

**Writing – review & editing:** Jun-Hong Guo.








