# Supplementary material for: Causal relationship between sex hormones and ischemic stroke risk: A Mendelian randomization study
Source: Medicine (Baltimore). 2026 Sep 4;105(36):e50336. doi: 10.1097/MD.0000000000050336 (PMC13549640; doi:10.1097/MD.0000000000050336)
Supplement: Supplementary file 1 [file medi-105-e50336-s001.docx]

**Table S1.** Data information used in this study

| **Phenotype** | | **Sample Size** | **Ancestry** |  |
| --- | --- | --- | --- | --- |
| Exposures | Sexual hormone binding globulin(SHBG) | 214,989Sample size/12,321,875Number of SNPs | European ancestry |  |
|  | Estradiol | 13,367cases/ 134,323 controls | European ancestry |  |
|  | Bioavailable testosterone | 382,988 Sample size/ 16,137,327 Number of SNPs | European ancestry |  |
|  |  |  |  |  |
| Outcomes | Ischemic stroke (IS) | 40,585 cases/406,111 controls | European ancestry |  |
|  | Large-artery atherosclerosis(LAA) | 4,373cases/406,111 controls | European ancestry |  |
|  | Small artery occlusion(SAO) | 5,386cases/192,662controls | European ancestry |  |
|  | Cardioembolism | 7,193cases/406,111controls | European ancestry |  |
