# Supplementary material for: Causal relationship between sex hormones and ischemic stroke risk: A Mendelian randomization study
Source: Medicine (Baltimore). 2026 Sep 4;105(36):e50336. doi: 10.1097/MD.0000000000050336 (PMC13549640; doi:10.1097/MD.0000000000050336)
Supplement: Supplementary file 2 [file medi-105-e50336-s002.docx]

**Table S2.** The characteristic information of instrumental variables for SHBG in MR analyses

| SNP | Chr | Position | EA | OA | Beta | | SE | | P-value | |
| --- | --- | --- | --- | --- | --- | --- | --- | --- | --- | --- |
| rs35504625 | 1 | 154599778 | G | C | 0.0299822 | 0.0029524 | | 3.10E-24 | |  |
| rs144613238 | 1 | 61693017 | A | AATTT | -0.0345806 | 0.00561056 | | 7.10E-10 | |  |
| rs28749241 | 1 | 115045665 | C | T | -0.0169493 | 0.00295465 | | 9.70E-09 | |  |
| rs9970073 | 1 | 214156165 | A | G | 0.0181328 | 0.00307794 | | 3.80E-09 | |  |
| rs3001032 | 1 | 219727779 | C | T | 0.0209467 | 0.00316475 | | 3.60E-11 | |  |
| rs469721 | 1 | 91530001 | T | C | -0.0259489 | 0.00372693 | | 3.30E-12 | |  |
| rs10922143 | 1 | 196821171 | A | G | 0.0241053 | 0.00312809 | | 1.30E-14 | |  |
| rs1223791 | 1 | 214321081 | A | G | -0.0289385 | 0.00399386 | | 4.30E-13 | |  |
| rs2486922 | 1 | 11124885 | C | T | -0.023057 | 0.0033718 | | 8.00E-12 | |  |
| rs114165349 | 1 | 27021913 | C | G | -0.146504 | 0.00984242 | | 4.10E-50 | |  |
| rs12121301 | 1 | 51038327 | A | T | 0.0187813 | 0.00297553 | | 2.80E-10 | |  |
| rs1730864 | 1 | 107607810 | T | C | -0.0425018 | 0.00313228 | | 6.10E-42 | |  |
| rs267733 | 1 | 150958836 | G | A | -0.0272953 | 0.00400964 | | 9.90E-12 | |  |
| rs16864042 | 1 | 171079005 | T | G | -0.0173313 | 0.00299143 | | 6.90E-09 | |  |
| rs10917373 | 1 | 23691745 | T | C | 0.0224874 | 0.00339598 | | 3.50E-11 | |  |
| rs201468966 | 1 | 39976059 | C | CA | 0.0266675 | 0.00351214 | | 3.10E-14 | |  |
| rs36209093 | 1 | 110229787 | T | C | -0.0250211 | 0.00350159 | | 9.00E-13 | |  |
| rs10801178 | 1 | 193119940 | C | G | 0.0185299 | 0.00318804 | | 6.20E-09 | |  |
| rs1260326 | 2 | 27730940 | C | T | 0.0637846 | 0.00301669 | | 3.10E-99 | |  |
| rs12613243 | 2 | 111897506 | C | T | -0.0355853 | 0.00606463 | | 4.40E-09 | |  |
| rs13427019 | 2 | 191564893 | A | T | -0.0195333 | 0.00348208 | | 2.00E-08 | |  |
| rs1047891 | 2 | 211540507 | A | C | 0.0256461 | 0.00316928 | | 5.90E-16 | |  |
| rs6758199 | 2 | 70537173 | T | C | -0.0454102 | 0.00595386 | | 2.40E-14 | |  |
| rs6736913 | 2 | 42510018 | G | A | -0.0649187 | 0.0104228 | | 4.70E-10 | |  |
| rs1014291 | 2 | 58884018 | T | G | 0.0178266 | 0.00298929 | | 2.50E-09 | |  |
| rs10184004 | 2 | 165508389 | T | C | 0.0330313 | 0.00300414 | | 4.00E-28 | |  |
| rs2160348 | 2 | 64909166 | C | T | -0.0560969 | 0.00336141 | | 1.60E-62 | |  |
| rs13423088 | 2 | 227026864 | A | T | 0.0290991 | 0.00317326 | | 4.70E-20 | |  |
| rs4518111 | 3 | 12377344 | C | A | 0.0258866 | 0.0029982 | | 5.90E-18 | |  |
| rs62271373 | 3 | 150066540 | A | T | -0.0456189 | 0.00634288 | | 6.40E-13 | |  |
| rs79287178 | 3 | 172294500 | A | G | -0.0543203 | 0.00897452 | | 1.40E-09 | |  |
| rs6792725 | 3 | 24520283 | G | A | 0.0349923 | 0.00329631 | | 2.50E-26 | |  |
| rs13086465 | 3 | 49779906 | T | C | -0.0277625 | 0.00316041 | | 1.60E-18 | |  |
| rs2289746 | 3 | 105455955 | C | T | 0.0176246 | 0.00314665 | | 2.10E-08 | |  |
| rs56298514 | 3 | 122359589 | C | A | -0.0198062 | 0.00346149 | | 1.10E-08 | |  |
| rs687339 | 3 | 135932359 | T | C | -0.0683786 | 0.00352857 | | 1.20E-83 | |  |
| rs724577 | 4 | 17993410 | C | A | -0.019965 | 0.00335904 | | 2.80E-09 | |  |
| rs3960788 | 4 | 103915618 | C | T | 0.0174139 | 0.00300405 | | 6.80E-09 | |  |
| rs28925904 | 4 | 144359490 | T | C | -0.0621911 | 0.00943409 | | 4.30E-11 | |  |
| rs6531735 | 4 | 39686332 | A | G | -0.0173749 | 0.00295808 | | 4.30E-09 | |  |
| rs2076947 | 4 | 148988159 | C | T | -0.0257823 | 0.00374068 | | 5.50E-12 | |  |
| rs2123885 | 4 | 69328988 | G | T | 0.0210116 | 0.00352624 | | 2.50E-09 | |  |
| rs7655064 | 4 | 120106348 | C | T | -0.0253791 | 0.00446946 | | 1.40E-08 | |  |
| rs76610881 | 4 | 159719955 | G | A | 0.0451543 | 0.0047491 | | 1.90E-21 | |  |
| rs13108218 | 4 | 3443931 | G | A | -0.0467657 | 0.00305625 | | 7.50E-53 | |  |
| rs1866975 | 4 | 77138658 | T | C | -0.022345 | 0.00304361 | | 2.10E-13 | |  |
| rs13150068 | 4 | 88203828 | G | A | -0.0329623 | 0.00298399 | | 2.30E-28 | |  |
| rs17547712 | 4 | 171180561 | A | G | 0.0321779 | 0.00553384 | | 6.10E-09 | |  |
| rs6860245 | 5 | 127367998 | C | G | 0.0252273 | 0.00343589 | | 2.10E-13 | |  |
| rs6865292 | 5 | 142792990 | C | T | -0.0187035 | 0.00337265 | | 2.90E-08 | |  |
| rs2963431 | 5 | 157970432 | T | C | -0.023049 | 0.0034808 | | 3.50E-11 | |  |
| rs255755 | 5 | 53307772 | G | A | -0.0245159 | 0.00335065 | | 2.50E-13 | |  |
| rs13179413 | 5 | 55868097 | T | C | -0.0228055 | 0.00333958 | | 8.60E-12 | |  |
| rs40270 | 5 | 55804552 | C | A | -0.038852 | 0.00352312 | | 2.80E-28 | |  |
| rs67570751 | 5 | 74974434 | C | CT | 0.0225895 | 0.00311022 | | 3.80E-13 | |  |
| rs806794 | 6 | 26200677 | G | A | 0.0239671 | 0.00330704 | | 4.30E-13 | |  |
| rs3130500 | 6 | 31119976 | A | T | -0.0269001 | 0.00338267 | | 1.80E-15 | |  |
| rs9766229 | 6 | 34408579 | C | A | 0.0433795 | 0.0075195 | | 8.00E-09 | |  |
| rs6939861 | 6 | 41703041 | A | G | -0.0374876 | 0.00340731 | | 3.70E-28 | |  |
| rs673736 | 6 | 160776653 | A | G | 0.0307208 | 0.00296955 | | 4.40E-25 | |  |
| rs998584 | 6 | 43757896 | A | C | -0.0211222 | 0.00296727 | | 1.10E-12 | |  |
| rs1293953 | 6 | 151991200 | C | G | 0.0214646 | 0.00323468 | | 3.20E-11 | |  |
| rs75479205 | 6 | 7255610 | G | A | 0.0254311 | 0.00378104 | | 1.70E-11 | |  |
| rs651837 | 6 | 139829560 | G | A | 0.0195602 | 0.0030061 | | 7.70E-11 | |  |
| rs740158 | 7 | 77055836 | C | T | 0.0162306 | 0.00296785 | | 4.50E-08 | |  |
| rs114949263 | 7 | 150498245 | C | T | 0.0304011 | 0.00471238 | | 1.10E-10 | |  |
| rs17884589 | 7 | 100493711 | A | G | 0.0355752 | 0.00380609 | | 9.00E-21 | |  |
| rs1229498 | 7 | 81568750 | G | T | -0.0215258 | 0.00335963 | | 1.50E-10 | |  |
| rs11556924 | 7 | 129663496 | T | C | 0.023141 | 0.00303607 | | 2.50E-14 | |  |
| rs157935 | 7 | 130585553 | G | T | 0.0179322 | 0.00322814 | | 2.80E-08 | |  |
| rs10622246 | 7 | 28187111 | A | ATTTT | -0.0168973 | 0.00296184 | | 1.20E-08 | |  |
| rs1688606 | 7 | 97974851 | A | G | 0.0742507 | 0.00381577 | | 2.40E-84 | |  |
| rs10238028 | 7 | 99208899 | G | A | 0.0396673 | 0.00590167 | | 1.80E-11 | |  |
| rs77258375 | 8 | 42332640 | C | CT | -0.0258712 | 0.00311175 | | 9.30E-17 | |  |
| rs12334548 | 8 | 72436958 | C | G | -0.0359285 | 0.00548636 | | 5.80E-11 | |  |
| rs5889386 | 8 | 11664542 | T | A | -0.0178382 | 0.00326265 | | 4.60E-08 | |  |
| rs2223054 | 8 | 116999486 | C | T | -0.0217112 | 0.00299089 | | 3.90E-13 | |  |
| rs9987289 | 8 | 9183358 | G | A | 0.0409459 | 0.00512214 | | 1.30E-15 | |  |
| rs10504255 | 8 | 59398461 | A | G | 0.0195318 | 0.00313626 | | 4.70E-10 | |  |
| rs150539196 | 8 | 81399180 | G | A | 0.0940289 | 0.00822247 | | 2.80E-30 | |  |
| rs5892750 | 8 | 81880253 | CA | C | 0.0327869 | 0.00369428 | | 7.00E-19 | |  |
| rs440837 | 8 | 81461974 | G | A | 0.0519732 | 0.00364977 | | 5.20E-46 | |  |
| rs13273326 | 8 | 145656806 | G | A | -0.0192839 | 0.00297287 | | 8.80E-11 | |  |
| rs62580785 | 9 | 113050798 | C | T | 0.0224057 | 0.00387333 | | 7.30E-09 | |  |
| rs4837794 | 9 | 123507855 | C | T | -0.0259469 | 0.00315623 | | 2.00E-16 | |  |
| rs112086410 | 9 | 131591894 | C | G | -0.0301626 | 0.00448813 | | 1.80E-11 | |  |
| rs7039093 | 9 | 5728192 | G | C | 0.0184145 | 0.00299864 | | 8.20E-10 | |  |
| rs28372209 | 9 | 92274389 | G | A | -0.0164395 | 0.00297218 | | 3.20E-08 | |  |
| rs2986670 | 9 | 113190709 | A | C | -0.017205 | 0.00315234 | | 4.80E-08 | |  |
| rs34463468 | 9 | 137267991 | T | C | -0.0310248 | 0.00357781 | | 4.30E-18 | |  |
| rs1330307 | 9 | 4305064 | C | A | -0.0174195 | 0.00297209 | | 4.60E-09 | |  |
| rs696825 | 9 | 86583076 | T | C | 0.0488535 | 0.00340666 | | 1.20E-46 | |  |
| rs62565259 | 9 | 102162570 | T | C | 0.023005 | 0.00395274 | | 5.90E-09 | |  |
| rs79717793 | 10 | 5262267 | A | G | -0.0421104 | 0.00409969 | | 9.50E-25 | |  |
| rs10824744 | 10 | 81094125 | G | A | -0.0280181 | 0.00371985 | | 5.00E-14 | |  |
| rs10822163 | 10 | 65124098 | G | C | 0.0920086 | 0.00296023 | | 1.00E-200 | |  |
| rs7921378 | 10 | 63674885 | C | G | -0.0202981 | 0.00296474 | | 7.60E-12 | |  |
| rs61850811 | 10 | 63914890 | A | G | 0.0310896 | 0.00499178 | | 4.70E-10 | |  |
| rs11188662 | 10 | 97961478 | T | C | 0.0196989 | 0.00315077 | | 4.00E-10 | |  |
| rs2068888 | 10 | 94839642 | A | G | 0.0237089 | 0.0029663 | | 1.30E-15 | |  |
| rs701810 | 10 | 98974849 | T | C | -0.0188615 | 0.00316889 | | 2.60E-09 | |  |
| rs2254069 | 10 | 122875589 | A | G | -0.0325153 | 0.00455527 | | 9.50E-13 | |  |
| rs11023881 | 11 | 16246700 | A | T | -0.0231105 | 0.00303378 | | 2.60E-14 | |  |
| rs10898075 | 11 | 82779736 | T | C | -0.0250661 | 0.00395441 | | 2.30E-10 | |  |
| rs174537 | 11 | 61552680 | T | G | -0.0276452 | 0.00311089 | | 6.30E-19 | |  |
| rs3015966 | 11 | 65217589 | C | T | -0.0238625 | 0.00313563 | | 2.70E-14 | |  |
| rs12805041 | 11 | 69284560 | T | C | 0.0328747 | 0.00351764 | | 9.10E-21 | |  |
| rs62618693 | 11 | 32956492 | T | C | 0.0432177 | 0.00716904 | | 1.70E-09 | |  |
| rs3842763 | 11 | 2179204 | T | G | 0.0277197 | 0.00349861 | | 2.30E-15 | |  |
| rs4938637 | 11 | 119075323 | A | G | 0.0357712 | 0.00560887 | | 1.80E-10 | |  |
| rs7947951 | 11 | 13356030 | G | A | -0.028028 | 0.00319489 | | 1.70E-18 | |  |
| rs12575636 | 11 | 95311260 | G | T | -0.0280622 | 0.00378802 | | 1.30E-13 | |  |
| rs1871395 | 12 | 21352315 | G | A | -0.0533039 | 0.00411869 | | 2.60E-38 | |  |
| rs11172134 | 12 | 57645789 | A | T | 0.0312034 | 0.00368476 | | 2.50E-17 | |  |
| rs7298698 | 12 | 103520884 | C | T | -0.0559534 | 0.00296513 | | 2.00E-79 | |  |
| rs377252458 | 12 | 69792102 | A | C | 0.019189 | 0.00320365 | | 2.10E-09 | |  |
| rs7304705 | 12 | 111438001 | G | A | 0.0553875 | 0.00549749 | | 7.10E-24 | |  |
| rs117233107 | 12 | 4328521 | A | G | 0.1107 | 0.0128532 | | 7.10E-18 | |  |
| rs11110390 | 12 | 100874901 | T | C | -0.0233781 | 0.00313808 | | 9.30E-14 | |  |
| rs141881700 | 12 | 101056213 | A | G | -0.119796 | 0.0133069 | | 2.20E-19 | |  |
| rs5009837 | 12 | 102810269 | C | T | -0.0276323 | 0.00327168 | | 3.00E-17 | |  |
| rs825453 | 12 | 124508758 | T | A | -0.0232519 | 0.00303328 | | 1.80E-14 | |  |
| rs11064358 | 12 | 6853766 | A | G | -0.0262817 | 0.00432125 | | 1.20E-09 | |  |
| rs1989390 | 12 | 21571656 | A | C | -0.023683 | 0.00402738 | | 4.10E-09 | |  |
| rs75130744 | 12 | 25410741 | C | G | -0.0530844 | 0.00577968 | | 4.10E-20 | |  |
| rs80127816 | 12 | 46267260 | A | G | -0.0384471 | 0.00666265 | | 7.90E-09 | |  |
| rs7310409 | 12 | 121424861 | G | A | 0.0271474 | 0.00304268 | | 4.60E-19 | |  |
| rs2130382 | 12 | 124484643 | G | C | 0.0206428 | 0.00319336 | | 1.00E-10 | |  |
| rs10841521 | 12 | 20587192 | G | A | 0.0185794 | 0.00338289 | | 4.00E-08 | |  |
| rs4307773 | 12 | 51144432 | C | T | -0.0264537 | 0.00299133 | | 9.30E-19 | |  |
| rs36179992 | 13 | 50639586 | G | GTA | -0.0196018 | 0.00303598 | | 1.10E-10 | |  |
| rs1239945 | 13 | 51107757 | A | G | -0.0162516 | 0.00297052 | | 4.50E-08 | |  |
| rs35792308 | 13 | 95234453 | C | CA | -0.019514 | 0.00326807 | | 2.40E-09 | |  |
| rs61755579 | 14 | 50655307 | T | C | 0.0549996 | 0.0086004 | | 1.60E-10 | |  |
| rs2239222 | 14 | 73011885 | G | A | 0.019216 | 0.00312118 | | 7.40E-10 | |  |
| rs13379043 | 14 | 74250126 | C | T | 0.0201121 | 0.00335821 | | 2.10E-09 | |  |
| rs17580 | 14 | 94847262 | A | T | 0.0459238 | 0.00695892 | | 4.10E-11 | |  |
| rs2498786 | 14 | 105262368 | G | C | -0.0226626 | 0.00304528 | | 9.90E-14 | |  |
| rs28929474 | 14 | 94844947 | T | C | 0.124662 | 0.0106081 | | 6.90E-32 | |  |
| rs34210072 | 14 | 23685442 | A | G | 0.0237447 | 0.00378804 | | 3.60E-10 | |  |
| rs11621792 | 14 | 24871926 | T | C | -0.0480818 | 0.0029971 | | 6.40E-58 | |  |
| rs144673482 | 15 | 41033280 | AAAAC | A | -0.0223375 | 0.0040488 | | 3.40E-08 | |  |
| rs8025155 | 15 | 53093869 | G | C | -0.0449663 | 0.00430213 | | 1.40E-25 | |  |
| rs79391862 | 15 | 53739426 | C | A | -0.148247 | 0.0128269 | | 6.80E-31 | |  |
| rs28539993 | 15 | 63790662 | T | C | 0.0266937 | 0.00299324 | | 4.70E-19 | |  |
| rs11637595 | 15 | 40387728 | T | C | -0.0186549 | 0.0033492 | | 2.50E-08 | |  |
| rs12914895 | 15 | 51054195 | G | C | 0.0166813 | 0.00297486 | | 2.10E-08 | |  |
| rs79240050 | 15 | 96667048 | A | G | 0.0580649 | 0.00915163 | | 2.20E-10 | |  |
| rs56332871 | 15 | 96714816 | A | C | 0.0749107 | 0.00332768 | | 3.20E-112 | |  |
| rs139974673 | 15 | 44027885 | C | T | -0.0834946 | 0.00923435 | | 1.50E-19 | |  |
| rs7194995 | 16 | 88013029 | A | C | -0.0277241 | 0.00352084 | | 3.40E-15 | |  |
| rs11644601 | 16 | 15172118 | C | T | 0.0240504 | 0.00323789 | | 1.10E-13 | |  |
| rs62033400 | 16 | 53811788 | G | A | -0.0236057 | 0.00303462 | | 7.30E-15 | |  |
| rs2925979 | 16 | 81534790 | C | T | 0.0253781 | 0.00323025 | | 4.00E-15 | |  |
| rs10083762 | 16 | 11907210 | G | C | 0.0194611 | 0.003323 | | 4.70E-09 | |  |
| rs12444108 | 16 | 30027694 | A | G | -0.0177667 | 0.00301912 | | 4.00E-09 | |  |
| rs7220103 | 17 | 47221643 | G | A | 0.0603862 | 0.00742767 | | 4.30E-16 | |  |
| rs72844546 | 17 | 73149850 | T | C | -0.0213563 | 0.00307746 | | 3.90E-12 | |  |
| rs191273168 | 17 | 7537135 | A | G | -0.192149 | 0.0109076 | | 1.90E-69 | |  |
| rs17880847 | 17 | 7573897 | A | T | -0.203969 | 0.0131101 | | 1.40E-54 | |  |
| rs80028739 | 17 | 7610490 | A | G | 0.129658 | 0.0157991 | | 2.30E-16 | |  |
| rs10153315 | 17 | 79481772 | C | T | -0.0191278 | 0.0029778 | | 1.30E-10 | |  |
| rs12947017 | 17 | 7318935 | T | C | 0.129396 | 0.00305543 | | 1.00E-200 | |  |
| rs117387630 | 17 | 7651906 | T | C | -0.307189 | 0.0105124 | | 1.00E-187 | |  |
| rs9911944 | 17 | 45498937 | T | G | -0.0403962 | 0.00293566 | | 4.40E-43 | |  |
| rs9907478 | 17 | 47395729 | T | C | 0.131801 | 0.00519306 | | 4.20E-142 | |  |
| rs142331290 | 17 | 7502034 | G | A | -0.172662 | 0.011754 | | 7.50E-49 | |  |
| rs140215187 | 17 | 7092533 | G | A | -0.0598337 | 0.00990605 | | 1.50E-09 | |  |
| rs57116414 | 17 | 7538323 | A | G | -0.149229 | 0.00843372 | | 4.60E-70 | |  |
| rs7224815 | 17 | 17845800 | T | A | -0.0326919 | 0.00298501 | | 6.50E-28 | |  |
| rs12943365 | 17 | 29680526 | G | C | 0.0215562 | 0.0030076 | | 7.70E-13 | |  |
| rs8178824 | 17 | 64224775 | T | C | -0.0816256 | 0.00858555 | | 2.00E-21 | |  |
| rs8087306 | 18 | 9094245 | C | G | -0.0189063 | 0.00319009 | | 3.10E-09 | |  |
| rs663640 | 18 | 57846077 | T | C | -0.022188 | 0.00357678 | | 5.50E-10 | |  |
| rs1217569 | 18 | 55081839 | A | G | -0.0241018 | 0.00366856 | | 5.00E-11 | |  |
| rs4804416 | 19 | 7223848 | G | T | -0.0361902 | 0.00298919 | | 9.70E-34 | |  |
| rs75702986 | 19 | 35566151 | A | G | 0.0295021 | 0.00383889 | | 1.50E-14 | |  |
| rs1350292 | 19 | 35553756 | T | C | 0.0640282 | 0.00591625 | | 2.70E-27 | |  |
| rs60321073 | 19 | 19499598 | G | A | -0.0240354 | 0.00386749 | | 5.10E-10 | |  |
| rs8104606 | 19 | 33888460 | A | G | 0.0175703 | 0.0031019 | | 1.50E-08 | |  |
| rs34255979 | 19 | 46384830 | T | C | 0.0511146 | 0.00455471 | | 3.20E-29 | |  |
| rs941408 | 19 | 2814181 | T | C | 0.0453878 | 0.0032662 | | 6.70E-44 | |  |
| rs202200760 | 19 | 17346854 | C | G | 0.155205 | 0.00830682 | | 6.70E-78 | |  |
| rs273492 | 19 | 18237882 | A | G | -0.0279686 | 0.00338989 | | 1.60E-16 | |  |
| rs74444983 | 19 | 45745607 | C | T | -0.020953 | 0.00339712 | | 6.90E-10 | |  |
| rs67611724 | 20 | 32308275 | T | C | -0.0249406 | 0.00408629 | | 1.00E-09 | |  |
| rs2618567 | 20 | 17844492 | T | G | -0.0193661 | 0.00312356 | | 5.60E-10 | |  |
| rs6129802 | 20 | 39920014 | T | C | 0.0235482 | 0.00360894 | | 6.80E-11 | |  |
| rs310670 | 20 | 62212392 | C | G | -0.0173515 | 0.00312717 | | 2.90E-08 | |  |
| rs6120663 | 20 | 33081906 | A | C | -0.0193988 | 0.0029854 | | 8.10E-11 | |  |
| rs55987409 | 20 | 49569025 | T | C | 0.0322976 | 0.00573092 | | 1.70E-08 | |  |
| rs6073431 | 20 | 43040569 | T | C | 0.0327618 | 0.00301919 | | 2.00E-27 | |  |
| rs6063803 | 20 | 51136424 | G | A | -0.0194566 | 0.00300595 | | 9.60E-11 | |  |
| rs4820091 | 22 | 21940189 | G | T | 0.0244305 | 0.00386379 | | 2.60E-10 | |  |
| rs757869 | 22 | 30764453 | A | G | -0.0306421 | 0.00325743 | | 5.10E-21 | |  |
| rs165722 | 22 | 19949013 | T | C | -0.0172758 | 0.00298459 | | 7.10E-09 | |  |
| rs3747207 | 22 | 44324855 | A | G | 0.0355567 | 0.00360423 | | 5.90E-23 | |  |

MR, Mendelian randomization; SNP, single nucleotide polymorphism; Chr, chromosome; EA, effect allele; OA,other allele, SE, standard error.
