# Supplementary material for: Causal relationship between sex hormones and ischemic stroke risk: A Mendelian randomization study
Source: Medicine (Baltimore). 2026 Sep 4;105(36):e50336. doi: 10.1097/MD.0000000000050336 (PMC13549640; doi:10.1097/MD.0000000000050336)
Supplement: Supplementary file 3 [file medi-105-e50336-s003.docx]

**Table S3.** The characteristic information of instrumental variables for [estradiol](javascript:;) in MR analyses

| SNP | Chr | Position | EA | OA | Beta | SE | P-value |
| --- | --- | --- | --- | --- | --- | --- | --- |
| rs112881196 | 2 | 31982811 | G | C | 0.302154 | 0.0300433 | 8.52E-24 |
| rs57102816 | 3 | 164506246 | TA | T | 0.0806118 | 0.0145276 | 2.87E-08 |
| rs7662029 | 4 | 69961912 | G | A | -0.112665 | 0.0129643 | 3.61E-18 |
| rs1073548 | 5 | 36012617 | G | T | 0.106682 | 0.0189315 | 1.75E-08 |
| rs45446698 | 7 | 99332948 | G | T | -0.209456 | 0.034576 | 1.38E-09 |
| rs600038 | 9 | 136151806 | C | T | -0.126217 | 0.0163807 | 1.31E-14 |
| rs56196860 | 12 | 2908330 | A | C | 0.233712 | 0.0337074 | 4.10E-12 |
| rs11160915 | 14 | 106514653 | A | G | -0.164053 | 0.0132441 | 3.08E-35 |
| rs3751591 | 15 | 51606710 | G | A | 0.107544 | 0.0168483 | 1.73E-10 |
| rs7173595 | 15 | 51533736 | T | C | 0.22874 | 0.0139251 | 1.24E-60 |
| rs62059839 | 17 | 7533015 | T | C | 0.0935541 | 0.0144126 | 8.53E-11 |
| rs113047993 | 18 | 20585399 | T | C | -0.153581 | 0.0271096 | 1.47E-08 |
| rs112285002 | 19 | 48374320 | T | C | 0.107149 | 0.0168783 | 2.18E-10 |

SNP, single nucleotide polymorphism; Chr, chromosome; EA, effect allele; OA, other allele; SE, standard error.
