# Supplementary material for: Causal relationship between sex hormones and ischemic stroke risk: A Mendelian randomization study
Source: Medicine (Baltimore). 2026 Sep 4;105(36):e50336. doi: 10.1097/MD.0000000000050336 (PMC13549640; doi:10.1097/MD.0000000000050336)
Supplement: Supplementary file 4 [file medi-105-e50336-s004.docx]

**Table S4.** The characteristic information of instrumental variables for Bioavailable testosterone in MR analyses

| SNP | Chr | Position | EA | OA | Beta | SE | P-value |
| --- | --- | --- | --- | --- | --- | --- | --- |
| rs114165349 | 1 | 27021913 | C | G | 0.053148 | 0.00663973 | 4.00E-16 |
| rs4655598 | 1 | 65932185 | A | T | -0.0111549 | 0.00200758 | 2.30E-08 |
| rs140584594 | 1 | 110232983 | G | A | 0.0166098 | 0.00223612 | 1.40E-13 |
| rs11240695 | 1 | 204158132 | A | C | 0.0183046 | 0.00231952 | 1.80E-15 |
| rs11588341 | 1 | 16498048 | G | A | -0.0120369 | 0.00202753 | 2.30E-09 |
| rs41264945 | 1 | 155640115 | T | C | 0.0244992 | 0.00437897 | 1.90E-08 |
| rs2248979 | 1 | 179310700 | T | C | 0.0159999 | 0.00231538 | 2.40E-12 |
| rs2392239 | 1 | 101735917 | T | C | 0.0193946 | 0.00217474 | 2.90E-19 |
| rs10192634 | 2 | 180500950 | T | C | 0.0166649 | 0.00225554 | 4.50E-13 |
| rs3832126 | 2 | 64906752 | CT | C | 0.0169961 | 0.00228885 | 5.50E-14 |
| rs590097 | 2 | 111934107 | G | T | 0.018975 | 0.00209302 | 1.10E-19 |
| rs13028479 | 2 | 11712075 | T | G | -0.0184182 | 0.00201877 | 3.20E-20 |
| rs35319517 | 2 | 43806172 | T | C | -0.0144524 | 0.00252126 | 4.70E-09 |
| rs113017476 | 2 | 31989359 | A | G | 0.0874817 | 0.00518438 | 7.80E-65 |
| rs2011425 | 2 | 234627608 | G | T | -0.0376561 | 0.00371428 | 8.90E-25 |
| rs11425772 | 3 | 88217876 | CA | C | -0.0150199 | 0.00264838 | 1.30E-08 |
| rs7610095 | 3 | 152035637 | A | G | -0.0160018 | 0.00200342 | 2.10E-15 |
| rs687339 | 3 | 135932359 | T | C | 0.023072 | 0.00238265 | 3.50E-22 |
| rs6771237 | 3 | 138247854 | A | T | -0.0122705 | 0.00200186 | 5.40E-10 |
| rs62260729 | 3 | 49889919 | C | T | 0.0136548 | 0.00213581 | 3.00E-10 |
| rs66956368 | 3 | 61279726 | A | T | -0.0167712 | 0.00230246 | 1.50E-13 |
| rs11919691 | 3 | 86782582 | G | A | -0.0132684 | 0.00205777 | 2.90E-10 |
| rs13065463 | 3 | 61662996 | A | G | -0.0169449 | 0.00296978 | 1.50E-08 |
| rs13108218 | 4 | 3443931 | G | A | 0.0176657 | 0.00207171 | 1.70E-17 |
| rs7679843 | 4 | 22028079 | G | C | 0.0235565 | 0.00349268 | 1.90E-11 |
| rs76229206 | 4 | 104797880 | C | T | -0.0339272 | 0.00536541 | 2.30E-10 |
| rs6854749 | 4 | 87968798 | T | A | 0.0144127 | 0.0024802 | 3.80E-09 |
| rs143039935 | 4 | 104578103 | AAAAACAAAAC | A | -0.0250353 | 0.00301011 | 9.80E-17 |
| rs115260227 | 4 | 104774698 | G | A | -0.111876 | 0.00962046 | 2.40E-31 |
| rs55644315 | 4 | 69945771 | GA | G | -0.0162315 | 0.00200448 | 2.50E-16 |
| rs12658172 | 5 | 124205385 | C | G | -0.018886 | 0.00277283 | 3.30E-12 |
| rs2312064 | 5 | 155807256 | G | C | -0.0111847 | 0.00201279 | 4.20E-08 |
| rs72834698 | 6 | 26176517 | A | G | -0.0177982 | 0.00286306 | 8.00E-11 |
| rs9378160 | 6 | 31467992 | C | A | 0.0148439 | 0.00253008 | 1.60E-10 |
| rs117108573 | 6 | 32489679 | T | C | 0.0209209 | 0.00391875 | 1.30E-08 |
| rs9495551 | 6 | 100236326 | T | C | -0.0170792 | 0.00241511 | 8.40E-13 |
| rs6912283 | 6 | 43364494 | A | G | 0.0138991 | 0.00201324 | 1.40E-12 |
| rs34192788 | 6 | 17416258 | A | T | -0.0130377 | 0.00218504 | 1.10E-09 |
| rs9322822 | 6 | 105369598 | T | C | -0.023054 | 0.00213857 | 8.90E-27 |
| rs1264332 | 6 | 30845563 | C | G | 0.0233666 | 0.00209381 | 2.20E-22 |
| rs9321953 | 6 | 144289022 | C | T | 0.0115038 | 0.00200797 | 6.20E-09 |
| rs794614 | 6 | 119284884 | G | A | -0.0229194 | 0.00241255 | 3.70E-22 |
| rs187383202 | 7 | 76357961 | T | C | -0.0533955 | 0.00919384 | 1.70E-08 |
| rs45446698 | 7 | 99332948 | G | T | -0.058755 | 0.00497617 | 1.20E-32 |
| rs10279715 | 7 | 40870935 | G | A | -0.014209 | 0.0020076 | 1.30E-12 |
| rs9986829 | 7 | 15019259 | A | G | 0.0279447 | 0.00200469 | 4.20E-43 |
| rs799158 | 7 | 73019074 | C | T | 0.0277953 | 0.00494232 | 2.40E-08 |
| rs4506131 | 7 | 97913196 | C | G | -0.0158816 | 0.00226765 | 1.40E-12 |
| rs7780066 | 7 | 137801915 | A | G | 0.0134951 | 0.00246504 | 3.30E-08 |
| rs4237037 | 8 | 61731073 | A | C | 0.0164615 | 0.00233007 | 6.70E-13 |
| rs284487 | 8 | 105957432 | T | C | 0.0119763 | 0.00213276 | 5.50E-09 |
| rs7006793 | 8 | 59461786 | C | T | 0.0125428 | 0.00215001 | 5.30E-09 |
| rs71529289 | 8 | 77879487 | T | C | -0.017019 | 0.00228128 | 1.80E-13 |
| rs9987289 | 8 | 9183358 | G | A | -0.0252994 | 0.00346152 | 2.00E-13 |
| rs6473232 | 8 | 81404297 | G | C | -0.0148692 | 0.00236934 | 2.00E-10 |
| rs13261573 | 8 | 25248615 | A | G | -0.0161763 | 0.00230981 | 2.30E-12 |
| rs1318995 | 9 | 24911674 | G | T | 0.0118184 | 0.00200463 | 3.70E-09 |
| rs10982156 | 9 | 117088064 | A | T | 0.0256267 | 0.00408608 | 3.70E-10 |
| rs10978435 | 9 | 108949159 | C | T | -0.0175351 | 0.00215378 | 4.80E-16 |
| rs61237993 | 9 | 34130435 | A | G | 0.0180937 | 0.00300057 | 9.40E-10 |
| rs12347838 | 9 | 108949596 | C | T | -0.0175311 | 0.0021541 | 5.10E-16 |
| rs148262335 | 9 | 77222516 | TTA | T | 0.017513 | 0.00212082 | 9.60E-17 |
| rs1937883 | 10 | 5062212 | A | G | -0.0182063 | 0.00220973 | 3.20E-16 |
| rs11441374 | 10 | 104619292 | AC | A | -0.024057 | 0.00233107 | 1.00E-25 |
| rs7924036 | 10 | 65191645 | T | G | -0.0165262 | 0.00199742 | 1.90E-16 |
| rs7912521 | 10 | 67262089 | C | T | 0.0306435 | 0.00202589 | 1.00E-51 |
| rs41313485 | 10 | 135055101 | A | G | 0.0333064 | 0.00568545 | 6.00E-09 |
| rs4565819 | 10 | 93646268 | A | G | -0.0158151 | 0.00265779 | 2.30E-09 |
| rs1994721 | 11 | 29204531 | A | G | -0.0335511 | 0.00282112 | 5.90E-33 |
| rs11389722 | 11 | 28077708 | GT | G | -0.0112928 | 0.00205942 | 2.80E-08 |
| rs12796488 | 11 | 94131557 | A | C | -0.0248549 | 0.00262245 | 3.70E-21 |
| rs10892924 | 11 | 122773715 | T | A | 0.0187203 | 0.00203107 | 3.00E-21 |
| rs112295236 | 11 | 62915346 | G | C | 0.0313604 | 0.00411368 | 2.30E-14 |
| rs7395670 | 11 | 66119563 | T | C | -0.0122007 | 0.00203825 | 2.80E-09 |
| rs7928369 | 11 | 125095221 | T | C | -0.0125316 | 0.00222955 | 6.20E-09 |
| rs7979473 | 12 | 121420260 | G | A | -0.017473 | 0.00206906 | 1.40E-17 |
| rs10745954 | 12 | 103483094 | G | A | 0.0129654 | 0.00199861 | 6.10E-11 |
| rs56196860 | 12 | 2908330 | A | C | 0.128389 | 0.00571671 | 4.80E-112 |
| rs4149056 | 12 | 21331549 | C | T | 0.0231508 | 0.0027858 | 2.20E-16 |
| rs2364232 | 12 | 93994827 | C | A | 0.0125666 | 0.00228639 | 3.20E-08 |
| rs4522206 | 12 | 130948847 | C | G | -0.0136065 | 0.00210549 | 6.50E-11 |
| rs76895963 | 12 | 4384844 | G | T | -0.065956 | 0.00770035 | 3.60E-18 |
| rs34138930 | 12 | 57359261 | CA | C | -0.0204126 | 0.00318294 | 2.90E-10 |
| rs629042 | 13 | 22318506 | C | G | 0.0173631 | 0.00205386 | 4.20E-18 |
| rs2038695 | 13 | 100559123 | A | C | -0.0113985 | 0.00202819 | 5.30E-09 |
| rs28929474 | 14 | 94844947 | T | C | -0.0915661 | 0.00714536 | 9.50E-39 |
| rs17580 | 14 | 94847262 | A | T | -0.0270762 | 0.00466701 | 9.90E-09 |
| rs73367233 | 14 | 106875386 | A | T | 0.0254333 | 0.00475235 | 4.30E-08 |
| rs11621792 | 14 | 24871926 | T | C | 0.01275 | 0.00201949 | 2.10E-10 |
| rs968257 | 14 | 64680285 | C | T | -0.0118628 | 0.00209955 | 2.20E-08 |
| rs2350892 | 14 | 60792103 | G | A | 0.014464 | 0.00207315 | 1.30E-12 |
| rs376117198 | 15 | 82426712 | CT | C | 0.0160784 | 0.00244195 | 1.40E-10 |
| rs56332871 | 15 | 96714816 | A | C | -0.0181845 | 0.0022547 | 8.50E-16 |
| rs7183977 | 15 | 40377092 | C | T | 0.0188057 | 0.00211228 | 7.80E-19 |
| rs967514 | 15 | 57413776 | G | A | 0.0115983 | 0.0020239 | 2.30E-08 |
| rs111475133 | 15 | 75810807 | TA | T | -0.0176792 | 0.00259395 | 1.40E-11 |
| rs17703883 | 15 | 51530097 | C | T | 0.0243257 | 0.00229818 | 3.60E-27 |
| rs2764772 | 16 | 20060653 | A | T | 0.0189214 | 0.00211997 | 8.80E-19 |
| rs1104608 | 16 | 73912588 | C | G | 0.0119033 | 0.00203942 | 5.80E-09 |
| rs12929087 | 16 | 9050639 | C | A | -0.0129587 | 0.00204457 | 1.50E-10 |
| rs76006132 | 16 | 81588571 | T | G | 0.0322757 | 0.00394023 | 1.10E-16 |
| rs8078625 | 17 | 1978334 | T | C | 0.0107762 | 0.00203675 | 4.50E-08 |
| rs727428 | 17 | 7537792 | C | T | -0.0642089 | 0.00201032 | 1.00E-200 |
| rs1050541 | 17 | 7560835 | G | T | -0.0304246 | 0.00206597 | 8.40E-49 |
| rs192287715 | 17 | 7261866 | A | G | 0.0297064 | 0.00370174 | 5.60E-16 |
| rs12940654 | 17 | 7268237 | G | A | -0.0255154 | 0.0036055 | 2.60E-13 |
| rs8178824 | 17 | 64224775 | T | C | 0.04606 | 0.00588661 | 5.80E-15 |
| rs10445374 | 17 | 45662383 | C | T | -0.0194906 | 0.00199961 | 4.80E-22 |
| rs11655191 | 17 | 47452263 | T | C | -0.0391797 | 0.00355831 | 1.30E-27 |
| rs2587507 | 17 | 77790135 | C | T | 0.0117181 | 0.00198261 | 2.90E-09 |
| rs2684839 | 18 | 44746947 | C | G | -0.0131626 | 0.00201536 | 3.80E-11 |
| rs73969326 | 18 | 71915785 | A | T | 0.0230056 | 0.00321217 | 1.20E-12 |
| rs202200760 | 19 | 17346854 | C | G | -0.0396479 | 0.00563831 | 8.30E-13 |
| rs73597479 | 19 | 35545385 | T | C | 0.0333661 | 0.00527443 | 1.10E-10 |
| rs7248104 | 19 | 7224431 | A | G | -0.0127354 | 0.00202954 | 1.10E-10 |
| rs7254216 | 19 | 48356741 | C | T | -0.0118109 | 0.00206389 | 9.90E-09 |
| rs7256062 | 19 | 49517293 | C | G | 0.0127754 | 0.00201851 | 1.80E-10 |
| rs201081113 | 20 | 33092419 | TC | T | -0.0121644 | 0.0019997 | 1.20E-09 |
| rs6142206 | 20 | 33212055 | A | G | 0.0133602 | 0.00202413 | 5.20E-11 |
| rs10212007 | 22 | 28560148 | G | C | 0.01963 | 0.00348949 | 3.10E-08 |
| rs6009583 | 22 | 49677646 | T | C | 0.018393 | 0.00226235 | 2.70E-16 |
| rs5978964 | X | 8744761 | T | C | 0.0162932 | 0.00175234 | 6.30E-21 |
| rs12397104 | X | 56612938 | T | C | 0.0358754 | 0.00647375 | 2.30E-08 |
| rs5965373 | X | 66409433 | G | T | 0.0232056 | 0.00236002 | 1.40E-22 |

SNP, single nucleotide polymorphism; Chr, chromosome; EA, effect allele; OA, other allele; SE, standard error.
